# Vitamin D Deficiency is Associated with Increased Disease Activity in Patients with Inflammatory Bowel Disease

**DOI:** 10.3390/jcm8091319

**Published:** 2019-08-27

**Authors:** Johannes Hausmann, Alica Kubesch, Mana Amiri, Natalie Filmann, Irina Blumenstein

**Affiliations:** 1 Department of Internal Medicine 1, Goethe-University Hospital Frankfurt, Theodor-Stern-Kai 7, 60590 Frankfurt, Germany; 2 Institute of Biostatistics and Mathematical Modeling, Goethe-University Hospital Frankfurt, Theodor-Stern-Kai 7, 60590 Frankfurt, Germany

**Keywords:** IBD, Crohn’s disease, Ulcerative colitis, intestinal barrier, mucosal inflammation, vitamin D, calcitriol

## Abstract

Background and Aims: Vitamin D has an inhibitory role in the inflammatory signaling pathways and supports the integrity of the intestinal barrier. Due to its immunomodulatory effect, vitamin D plays a role in chronic inflammatory bowel disease (IBD) and a deficiency is associated with an increased risk for a flare. We aimed to investigate to what extent the 25-hydroxyvitamin D (25(OH)D_3_) level correlates with disease activity and whether a cut-off value can be defined that discriminates between active disease and remission. Methods: Patients with IBD, treated at the University Hospital Frankfurt were analyzed retrospectively. The 25(OH)D3 levels were correlated with clinical activity indices and laboratory chemical activity parameters. A deficiency was defined as 25(OH)D3 levels <30 ng/mL. Results: A total of 470 (257 female) patients with IBD were included, 272 (57.9%) with Crohn’s disease (CD), 198 (42.1%) with ulcerative colitis (UC). The median age of the patients was 41 (18–84). In 283 patients (60.2%), a vitamin D deficiency was detected. 245 (53.6%) patients received oral vitamin D supplementation, and supplemented patients had significantly higher vitamin D levels (*p* < 0.0001). Remission, vitamin D substitution, and male gender were independently associated with the 25(OH)D3 serum concentration in our cohort in regression analysis. A 25(OH)D3 serum concentration of 27.5 ng/mL was the optimal cut-off value. Conclusion: Vitamin D deficiency is common in IBD patients and appears to be associated with increased disease activity. In our study, vitamin D levels were inversely associated with disease activity. Thus, close monitoring should be established, and optimized supplementation should take place.

## 1. Introduction

Intestinal barrier dysfunction plays a key role in the pathogenesis of inflammatory bowel disease (IBD) as it leads to the activation of the inflammatory signaling pathways, ultimately causing intestinal inflammation [1]. Despite being most prominently known as an essential hormone in bone metabolism regulation, vitamin D—a pleiotropic hormone [2]—does also play a crucial role in the regulation of the immune response [3,4,5]. In the gastrointestinal tract, vitamin D is involved in the gut immunity and microbiota, as well as in the intestinal epithelial barrier function [6,7]. Furthermore, studies have shown that vitamin D does have an influence on bacterial composition in Crohn’s disease (CD) [8].

The vitamin D receptor (VDR)—serving as the mediator for Calcitriol (1,25(OH)_2_D_3_)—is abundantly expressed in the intestine. However, the role of VDR signaling in the gut is—to date—not entirely understood. One ascribed function is maintaining the integrity of the intestinal barrier by promoting tight junction proteins such as claudin-1, and thus strengthening the integrity of intestinal epithelial cells [9,10,11]. There is a growing body of evidence that other micronutrients or vitamins—for instance vitamin A—do also play a significant role in the inflammatory response [12].

Studies have shown that vitamin D deficiency may increase the risk IBD and has a high prevalence in IBD patients throughout all disease stages (i.e., flare and remission) [13,14]. A recent systematic review and meta-analysis reported a prevalence of 38.1% and 31.6% for vitamin D deficiency for Crohn’s disease (CD) and Ulcerative colitis (UC), respectively [15] as well as higher odds for developing a vitamin D deficiency compared with controls. Furthermore, studies have shown that vitamin D deficiency is associated with increased disease activity [14,16], and another study from the United States even determined vitamin D deficiency to be independently associated with greater disease activity in IBD and lower quality of life [13]. In light of the growing evidence of vitamin D’s possible beneficial effects in IBD disease activity, vitamin D supplementation—if indicated—is recommended by the respective guideline of the European Society for Clinical Nutrition and Metabolism (ESPEN) [17]. However, the optimal vitamin D level, supplementation modality, as well as vitamin D deficiency’s association with clinical parameters relevant to IBD are still poorly defined. The aim of this retrospective cohort study was to define the optimal vitamin D serum level cut-off and parameters associated with vitamin D deficiency.

## 2. Patients and Methods

### 2.1. Study Population

In this retrospective single-center study, all patients being treated in the IBD outpatient clinic of the University Hospital Frankfurt, Germany with IBD between 01.01.2017 and 31.12.2018 were included. Inclusion criteria were age above 18 years, and diagnosed IBD (either UC or CD). Exclusion criteria were age below 18 years and no definitively diagnosed IBD. At the time of inclusion, 25(OH)D3 serum levels as well as clinical and laboratory variables reflecting disease activity, vitamin D supplementation regimen, and patient characteristics were determined. The patients were followed up at the treating physician’s discretion, at least every six months to monitor their IBD treatment. 25(OH)D3 serum levels were only measured every 12 months. Approval for this retrospective study was obtained from the local Ethics Committee of the University Hospital Frankfurt (file number 414/18, approved on 29 November 2018).

### 2.2. Quantification of 25-hydroxyvitamin D (25(OH)D_3_) Serum Levels and Other Laboratory Parameters

All laboratory parameters were measured in the central laboratory of the University Hospital Frankfurt. 25(OH)D_3_ levels were defined as follows: levels ≥30 ng/mL were considered as sufficient, 25(OH)D_3_ levels between 10 and 29 ng/mL and <10 ng/mL were considered as deficient and severely deficient, respectively [2].

### 2.3. Disease Activity and IBD-related Medication

Disease activity was monitored with the help of the Harvey–Bradshaw Index (HBI) and the Simple Clinical Colitis Activity Index (SCCAI), both well-established tools to monitor IBD activity [18,19,20]. A HBI score of <5 was considered a state of remission, whereas scores ≥5 were defined as active disease. A SCCAI score of ≥5 was defined as active disease [21]. For the regression analysis, disease activity was summarized into remission (i.e., HBI < 5 or SCCAI < 5) or active disease (i.e., HBI ≥ 5 or SCCAI ≥ 5). Information on IBD specific medication was derived from the patient chart. For the regression analysis, medication was divided into conventional therapy (i.e., Azathioprine, Mesalamine, Steroid, and other therapies) and biological therapy (tumor necrosis factor α (TNFα) inhibitors, integrine inhibitors, and Ustekinumab).

### 2.4. Statistical Analyses

Statistical analyses were conducted using IBM SPSS Statistics Version 22.0 (International Business Machine Corporation, Endicott, NY, USA) and BiAS, Version 11.02. Group differences were assessed by means of χ^2^ contingency tables or Wilcoxon–Mann–Whitney U tests, as appropriate. *P* values ≤ 0.05 were considered to be statistically significant. All tests are two-sided. Associations of outcomes with dichotomic variables were assessed in binary logistic regression models, respectively. After univariate analyses, multivariate analyses were performed for significant associations. Multivariate models were obtained by backward selection, using a *p* value >0.1 for removal from the model. The cut-off value for vitamin D was also evaluated by calculating a Receiver-Operating-Characteristics (ROC) analysis (using patients in remission as negative outcome).

## 3. Results

### 3.1. Patient Characteristics

A total of 470 patients with IBD were included in this retrospective cohort study. Of the entire cohort, 272 (57.9%) suffered from CD, whereas as 198 (42.1%) suffered from UC. 257 (54.7%) were male, and the median age was 41 years (range 18–84 years). 283 patients (60.2%) had a relevant vitamin D deficiency. Patients with CD were significantly younger (*p* = 0.027) and had higher C-reactive Protein (CRP) (*p* = 0.049) and leukocyte (*p* = 0.012) levels, and more often received biological treatment than patients with UC. The characteristics of the included patients are shown in Table 1.

### 3.2. 25(OH)D_3_ Serum Concentrations and Associated Seasonal Variations

The median 25(OH)D_3_ serum concentration of the entire study population was 26 ng/mL. Two hundred eighty-three (60.2%) patients had a deficiency—defined as 25(OH)D_3_ serum levels below 30 ng/mL. Patients with CD had significantly lower median 25(OH)D_3_ serum levels than patients with UC (25.5 (3–76) vs. 28 (3–100), *p* = 0.049). Two hundred forty-five patients (53.6%) received a vitamin D supplementation (mainly 20000 IU cholecalciferol once weekly and in a few cases 1000 IU cholecalciferol daily), and in 112 (45.7%) patients, a relevant vitamin D deficiency was still present despite the supplementation. Overall, in patients receiving supplementation, the median 25(OH)D_3_ serum concentration was significantly higher (31 ng/mL (range 4–100)) in comparison with patients without supplementation (22 ng/mL (range 3–70)) (*p* < 0.0001). In the entire cohort, sufficient 25(OH)D_3_ serum levels (≥30 ng/mL) were observed in 188 patients (40%), moderate (10–30 ng/mL) and severe deficiency (<10 ng/mL) in 232 (49.4%) and 49 (10.4%) patients, respectively. Out of the patients with severely low 25(OH)D_3_ serum concentrations, 13 (27.7%) received supplementation. Interestingly, median 25(OH)D_3_ serum levels were significantly lower in patients with active disease in comparison with patients in remission (23 ng/mL (4–75) vs. 29 ng/mL (3–100), *p* = 0.04) (Table 2).

Concerning seasonal variations of 25(OH)D_3_, several significant differences could be observed. In 303 cases, serum vitamin D levels were measured in the winter season (i.e., between October and March), whereas in 167 cases, levels were assessed in the summer season (i.e., between April and September). Patients in the summer group had significantly higher vitamin D serum levels. This was observed for the entire cohort (*p* = 0.002) as well as for the subgroups (CD *p* = 0.009 and UC *p* = 0.04). Patients with oral vitamin D supplementation had significantly higher serum levels in both seasons; this was observed for the entire cohort (*p* < 0.0001) as well as for the CD subgroup (*p* < 0.0001). Interestingly, for patients with UC, highly significant results concerning vitamin D substitution were only observed in the winter season (*p* < 0.0001), whereas in the summer season, the difference was less significant (*p* = 0.02). For a detailed overview please see Supplementary Table S1.

### 3.3. IBD-related Medication

Details on IBD-related medication in our cohort are provided in Table 3 of the manuscript. Statistically significant (*p* < 0.0001), more patients with CD received biological treatment, whereas patients in the UC cohort were more likely to receive conventional treatment.

### 3.4. Logistic Regression Analysis for Parameters Associated with Vitamin D Deficiency

Univariate and multivariate linear regression analyses were performed to determine parameters possibly associated with vitamin D serum levels in our patient collective. The analyses were conducted separately for the entire cohort as well as for the subgroup of patients with CD and UC. Parameters evaluated included: CRP, leukocyte levels, vitamin D supplementation, biological therapy, gender, fecal calprotectin (fCal), and the disease activity, defined as either remission (HBI < 5 or SCCAI < 5) or active disease (HBI ≥ 5 or SCCAI ≥ 5).

In the multivariate analysis, for the entire cohort, remission (multivariate *p* = 0.02, odds ratio (OR) = 2.01 (95% confidence interval (CI) = 1.10–3.69)), vitamin D supplementation (multivariate *p* < 0.0001, OR = 4.64 (95% CI = 2.56–8.39)), and male gender (multivariate *p* = 0.06, OR = 1.69 (95% CI = 0.96–2.97)) were independently associated with low 25(OH)D_3_ serum. In the subgroup analysis of patients with CD, fCal (multivariate *p* = 0.05, OR = 0.99 (95% CI = 0.99–1.00)), biological therapy (multivariate *p* = 0.01, OR = 0.35 (95% CI = 0.15–0.81)), remission (multivariate *p* = 0.05, OR = 2.11 (95% CI = 0.97–4.56)), and vitamin D supplementation (multivariate *p* < 0.0001, OR = 4.37 (95% CI = 1.91–10.00)) were independently associated with low 25(OH)D_3_ serum levels. For remission and vitamin D supplementation, an inverse association was observed. Interestingly, in the UC group, male gender (multivariate *p* = 0.003, OR = 2.70 (95% CI = 1.40–5.18)), age (multivariate *p* = 0.04, OR = 1.02 (95% CI = 1.02–1.04)), and vitamin D supplementation (multivariate *p* < 0.0001, OR = 4.35 (95% CI = 2.23–8.47)) were independently associated with vitamin D deficiency (Table 4).

### 3.5. ROC Analysis to Determine 25(OH)D_3_ Serum Concentration CutOff

**Figure 1:**
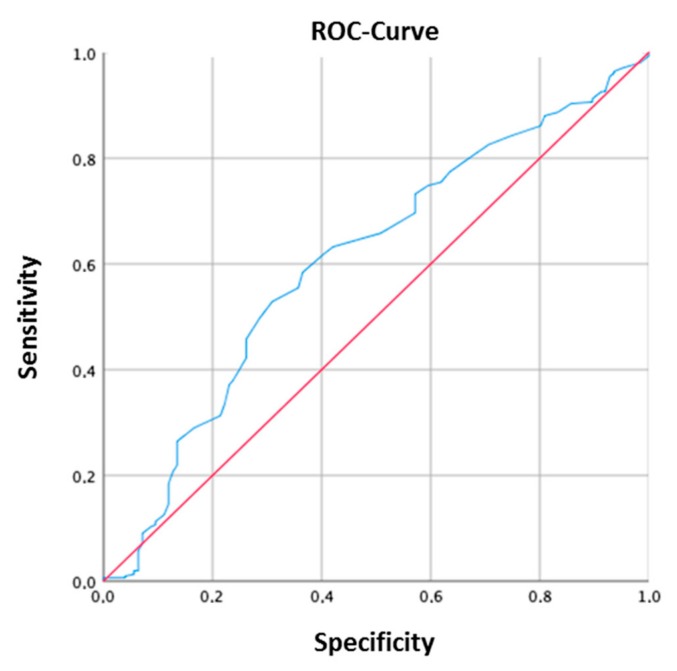
Area under the receiver operating characteristic curve (AUROC) of vitamin D serum levels predicting the risk of flare in IBD patients. A 25-hydroxyvitamin D (25(OH)D_3_) serum concentration of 27.5 ng/mL was identified as the optimal cut off.

The area under the receiver operating characteristic curve (AUROC) was calculated for the entire cohort. The ROC analysis showed that a 25(OH)D_3_ serum concentration of 27.5 ng/mL is the optimal cut-off to discriminate between active disease and remission in our patients (Figure 1).

## 4. Discussion

Vitamin D deficiency is a frequent finding in healthy worldwide populations [22]. Over the past decade, the immunomodulatory effect of vitamin D has become of great interest, especially for IBD. Thus, understanding the possible negative implications of a vitamin D deficiency with regard to the natural history of the disease and its activity has become increasingly relevant. Vitamin D deficiency is more frequent in patients with IBD [23], and several studies were able to show that supplementation leads to an improvement of the disease activity [13,24,25]. Although it is generally agreed upon that vitamin D levels within the normal range should be the goal and, if need be, achieved by supplementation, there still is no general consensus what serum vitamin D level constitutes a deficiency, and how vitamin D supplementation and monitoring should be conducted. Measuring 25(OH)D3 serum levels is considered to be the most accurate way to determine the general vitamin D status in the body [26]. However, “cut-off” values do vary widely depending on the respective field and the ascribed role of vitamin D (i.e., bone metabolism or immunomodulation). Endocrinologists, for instance, consider serum level below 20 ng/mL (i.e., 50 nmol/L) to be a vitamin D deficiency, though it is believed that the cut-off for vitamin D’s immunomodulatory benefits is higher, at around 30 ng/mL (i.e., 75 nmol/L) [26]. Since multiple studies have shown that low serum vitamin D levels are injurious for IBD patients [16,25,26,27], it is crucial to determine potential cut-off values to best reap vitamin D’s potential benefits in patients with IBD.

With this in mind, we sought to determine if a cut-off value that discriminates between active disease and remission could be defined and if 25(OH)D3 levels are associated with clinical and laboratory parameters of disease activity in our cohort. Included in the study were 463 (257 female, median age 41 (18–84)) patients being treated for IBD in a tertiary care center. Of our study population, 281 (60.7%, 163 female, median age 39 (18–84)) had a vitamin D deficiency. In literature reports, incidence of vitamin D deficiency vary—a Korean study reported an incidence of 89.2%, whereas a retrospective American study reported one of only 49.8%. Interestingly, in our cohort, patients with CD (*n* = 272, 57.9%) had significantly lower 25(OH)D_3_ serum levels (*p* = 0.049), were significantly younger (*p* = 0.027), and had higher CRP (*p* = 0.049) and leukocyte (*p* = 0.012) levels than patients with UC. Furthermore, patients with CD were more likely to receive biological therapy, possibly highlighting a higher disease activity in these patients. The observed tendency that vitamin D deficiency is more common in patients with CD has been reported in several studies [28,29]. Patients receiving supplementation had significantly higher vitamin D levels (31 ng/mL vs. 22 ng/mL (*p* < 0.0001) and, interestingly, of the supplemented patients, 13 (27.7%) still had a severe vitamin D deficiency despite supplementation. The observed persisting deficiency under supplementation in our cohort might indicate that supplemented patients had lower initial serum levels and that the standard supplementation regimen is obviously not sufficient in these cases. Thus, adjustments to the vitamin D supplementation and monitoring regimen should be considered. Data from a prospective study from Ireland supports this observation. Here, 43% of patients with CD already under vitamin D supplementation still had a deficiency. In this study, supplementation usually constituted of a low-dose vitamin D supplement, 200–400 IU/day [30]. In our cohort, patients with a vitamin D deficiency received 20.000 IU (oral) of vitamin D daily for 10 days, followed by 20.000 IU of vitamin D per week. Furthermore, we observed that in the winter season, vitamin D deficiency was more frequent in our cohort. This is in line with a recently published article by Janssen et al. [31].

With focus on the clinical and laboratory parameters of disease activity, vitamin D supplementation, male gender, and biological therapy were independently associated with a low serum 25(OH)D_3_ in our cohort. In the CD group, the HBI, fCal, biological therapy, and vitamin D supplementation were independently associated with low 25(OH)D_3_ serum levels. However, in the UC group, slight differences could be observed. Here male gender, age, and vitamin D supplementation were independently associated with vitamin D deficiency. Both vitamin D supplementation and remission were inversely associated with vitamin D deficiency. A possible explanation for differences in the subgroup analyses might be that patients in the UC group were more likely to be in remission, and fewer patients received biological treatment. A recent American study was also able to show that low serum 25(OH)D_3_ levels are associated with increased disease activity in patients with CD [25]. Interestingly, a Norwegian study showed that vitamin D deficiency in patients with UC was not associated with the disease activity (SCCAI) but with elevated fCal levels [29]. In our study, markers of systemic inflammation (i.e., CRP) were not associated with vitamin D deficiency. Interestingly, Garg et al. were able to show that vitamin D can reflect inflammation in local tissue but not necessarily systemic inflammation [32].

Furthermore, we were able to determine that a 25(OH)D_3_ serum level of 27.5 ng/mL was the optimal cut-off to discriminate between active disease and remission in our patients with IBD. Another German cohort study in IBD patients determined a cut-off value of 19 ng/mL to discriminate between clinical remission and increased disease activity in patients with CD [33]. The variance in cut-off values between our cohort and the one of Mechie et al. might be due to the fact that over 70% of the patients in our cohort were in remission and that the median vitamin D levels were also significantly higher.

Limitations to our study are limited specific baseline and no follow-up data, due to the retrospective nature of the study. However, results from two prospective trials from Norway and the US also showed an inverse association of vitamin D and disease activity, and thus support our findings [25,29]. We do believe that our study has its merits, as we provide data on vitamin D deficiency in a large northern European cohort, which is possibly more susceptible to vitamin D deficiency due to the climate and low sun exposure.

## 5. Conclusions

To conclude, our study provides insights on vitamin D deficiency in a large German cohort and defines a cut-off value to discriminate between active disease and remission, which could be especially relevant for patient monitoring and future prospective trials.

## Figures and Tables

**Table 1:** Patient characteristics and group differences.

Parameters	Entire Cohort *n* = 470	Ulcerative Colitis *n* = 198	Crohn’s Disease *n* = 272	*p* Value
Female sex, *n* (%)	257 (54.7)	94 (47.5)	163 (59.9)	
Age, median (range)	41 (18–84)	45 (18–84)	39 (18–84)	*p* = 0.027
Remission ^#^	312 (71.6)	161 (83.9)	151 (61.9)	*p* < 0.0001
HBI, mean (SD)	N/A	N/A	4.23 (3.56)	
SCCAI, mean (SD)	N/A	2.15 (2.46)	N/A	
CRP mg/dL, mean (SD)	0.66 (1.2)	0.55 (0.9)	0.74 (1.3)	*p* = 0.049
Leukocytes/nL, mean (SD)	8.17 (2.9)	7.88 (2.9)	8.38 (2.8)	*p* = 0.012
Biological Therapy *	199 (53.4)	54 (32.9)	145 (69.4)	*p* < 0.0001
25(OH)D_3_ (ng/mL); median (range)	26 (3–100)	28 (3–100)	25.5 (3–76)	*p* = 0.049
Vit D supplementation	245 (53.6)	108 (55.7)	137 (52.1)	
Vit D measured in winter	303 (64.5)	139 (70.2)	164 (60.3)	
fCal µg/g, mean (SD)	315 (352)	356 (367)	289 (341)	

Harvey–Bradshaw Index (HBI), Simple Clinical Colitis Activity Index (SCCAI), C-reactive Protein (CRP), Fecal Calprotectin (fCal), ^#^ for 436 patients, disease activity was provided, remission was defined as HBI < 5 and SCCAI < 5 * Biological therapy defined as treatment with TNFα Inhibitor, Integrin Inhibitor, or Ustekinumab, detailed information is provided below.

**Table 2:** 25-hydroxyvitamin D (25(OH)D_3_) serum levels in inflammatory bowel disease (IBD) patients.

25(OH)D_3_ Serum Levels ng/mL	Number of Patients
**Overall (*n* = 470)**	(%)
25(OH)D_3_ ≥ 30 ng/mL, *n* (%)	188 (40)
25(OH)D_3_ 30–10 ng/mL, *n* (%)	232 (49.4)
25(OH)D_3_ < 10 ng/mL, *n* (%)	49 (10.4)
**Crohn’s disease (*n* = 272)**	
25(OH)D_3_ ≥ 30 ng/mL, *n* (%)	99 (36.4)
25(OH)D_3_ 30–10 ng/mL, *n* (%)	144 (52.9)
25(OH)D_3_ < 10 ng/mL, *n* (%)	28 (10.3)
**Ulcerative colitis (*n* = 198)**	
25(OH)D_3_ ≥ 30 ng/mL, *n* (%)	89 (44.9)
25(OH)D_3_ 30–10 ng/mL, *n* (%)	88 (44.4)
25(OH)D_3_ < 10 ng/mL, *n* (%)	21 (10.6)

**Table 3:** Inflammatory Bowel Disease (IBD)-related Medication.

Medication	Entire Cohort	Ulcerative Colitis	Crohn’s Disease
**Conventional Treatment**	169 (36)	110 (55.6)	64 (30.6)
5-ASA total, *n* (%)	86 (23)	75 (37.8)	17 (6.3)
5-ASA, *n* (%)	74 (19.8)	57 (28.8)	17 (6.3)
5-ASA in combination, *n* (%)	12 (3.2)	18 (9)	N/A
Glucocorticoids, *n* (%)	21 (5.6)	9 (4.5)	12 (4.4)
Azathioprine/6-MP total, *n* (%)	52 (13.9)	21 (10.6)	31 (11.7)
Azathioprine/6-MP, *n* (%)	38 (10.2)	12 (6.1)	26 (9.8)
Azathioprine/6-MP in combination, *n* (%)	14 (3.7)	9 (4.5)	5 (1.9)
Other Therapies, *n* (%)	10 (2.7)	5 (2.5)	4 (1.5)
**Biological treatment**	198 (42.1)	51 (25.7)	140 (50)
TNFα Inhibitor total, *n* (%)	105 (28.1)	22 (11.1)	78 (29.5)
TNFα Inhibitor, *n* (%)	77 (20.6)	10 (5)	62 (23.4)
TNFα Inhibitor in combination, *n* (%)	28 (7.5)	12 (6.1)	16 (6.1)
Integrin Inhibitor total, *n* (%)	65 (17.4)	29 (14.6)	36 (13.6)
Integrin inhibitor, *n* (%)	51 (13.7)	20 (10)	32 (12.1)
Integrin inhibitor in combination, *n* (%)	14 (3.7)	9 (4)	4 (1.5)
Il12/23 blocker, *n* (%)	26 (6.9)	N/A	26 (9.6%)
**No treatment**	90 (19.1)	28 (14.4)	62 (22.8)
**Missing information**	13 (2.8)	9 (4.5)	6 (2.3)

5-aminosalicylic acid (5-ASA), 6-mercaptopurine (6-MP), tumor necrosis factor α (TNFα), interleukine (IL).

**Table 4:** Logistic regression analysis for factors associated with 25-hydroxyvitamin D (25(OH)D_3_) serum levels (<30ng/mL) in Inflammatory Bowel Disease (IBD) patients.

	Univariate Analysis		Multivariate Analysis	
	*p* Value	OR (95% CI)	*p* Value	OR (95% CI)
**Entire cohort**				
Age	0.007	1.01 (1.00–1.03)		
Male Gender	0.05	1.44 (0.96–2.09)	0.06	1.69 (0.96–2.97)
Remission (yes)	0.0001	2.38 (1.56–3.64)	0.02	2.01 (1.10–3.69)
Leukocytes	0.049	0.93 (0.87–0.99)		
fCal µg/g	0.042	0.99 (0.99–1.00)		
Vit. D supplementation (yes)	0.0001	3.74 (2.50–5.61)	0.0001	4.64 (2.56–8.39)
Biological therapy (yes)	0.032	0.63 (0.42–0.96)		
**Crohn’s disease**				
Remission (yes)	0.001	2.51 (1.47–4.28)	0.05	2.11 (0.97–4.56)
fCal µg/g	0.03	0.99 (0.99–1.00)	0.05	0.99 (0.99–1.00)
Vit. D supplementation (yes)	0.0001	3.72 (2.16–6.39)	0.0001	4.37 (1.91–10.00)
Biological therapy (yes)	0.009	0.45 (0.24–0.82)	0.01	0.35 (0.15–0.81)
**Ulcerative colitis**				
Age	0.006	1.02 (1.00–1.05)	0.04	1.02 (1.00–1.04)
Male Gender	0.020	1.98 (1.20–3.50)	0.003	2.70 (1.40–5.18)
Remission (yes)	0.07	2.13 (0.92–4.91)		
Vit. D supplementation (yes)	0.0001	3.75 (2.04–6.90)	0.0001	4.35 (2.23–8.47)

Odds ratio (OR), confidence interavl (CI), fecal calprotectin (fCal).

## Supplementary Materials

The following are available online at https://www.mdpi.com/2077-0383/8/9/1319/s1, Table S1: Seasonal variations in vitamin D serum levels.
