# Supplementary material for: Vitamin D Deficiency is Associated with Increased Disease Activity in Patients with Inflammatory Bowel Disease
Source: J Clin Med. 2019 Aug 27;8(9):1319. doi: 10.3390/jcm8091319 (PMC6780251; doi:10.3390/jcm8091319)
Supplement: Supplementary file 1 [file jcm-08-01319-s001.pdf]

Table S1. Seasonal variations in vitamin D serum levels.

	Entire cohort	Ulcerative colitis	Crohn's disease
**Winter season (i.e. Oct–Mar), *n***			
Overall value, *n*; median (range)	303 patients<br>24 (3–75)	134 patients<br>25 (3–75)	164 patients<br>23 (4–70)
Without substitution, median (range)	134 patients<br>21 (3–70)	57 patients<br>21 (3–47)	77 patients<br>21 (4–70)
With substitution, median (range)	160 patients<br>29 (4–75)	79 patients<br>31 (4–75)	81 patients<br>28 (5–64)
**Summer season (i.e. Apr–Sep), *n***			
Overall value, median (range)	167 patients<br>29 (3–100)	59 patients<br>31 (5–100)	108 patients<br>27 (3–76)
Without substitution, median (range)	78 patients<br>26 (3–52)	29 patients<br>28 (5–52)	49 patients<br>26 (3–49)
With substitution, median (range)	85 patients<br>34 (6–100)	29 patients<br>35 (8–100)	56 patients<br>32.5 (6–76)
